# Robust Genome Editing with Short Single-Stranded and Long, Partially Single-Stranded DNA Donors in *Caenorhabditis elegans*

**DOI:** 10.1534/genetics.118.301532

**Published:** 2018-09-13

**Authors:** Gregoriy A. Dokshin, Krishna S. Ghanta, Katherine M. Piscopo, Craig C. Mello

**Affiliations:** * RNA Therapeutics Institute, University of Massachusetts Medical School, Worcester, Massachusetts 01605; † Howard Hughes Medical Institute, Worcester, Massachusetts 01605

**Keywords:** CRISPR, HDR, fluorescent tags, WormBase

## Abstract

CRISPR-based genome editing using ribonucleoprotein complexes and synthetic single-stranded oligodeoxynucleotide (ssODN) donors can be highly effective. However, reproducibility can vary, and precise, targeted integration of longer constructs—such as green fluorescent protein tags remains challenging in many systems. Here, we describe a streamlined and optimized editing protocol for the nematode *Caenorhabditis elegans*. We demonstrate its efficacy, flexibility, and cost-effectiveness by affinity-tagging 14 Argonaute proteins in *C. elegans* using ssODN donors. In addition, we describe a novel PCR-based, partially single-stranded, “hybrid” donor design that yields high efficiency editing with large (kilobase-scale) constructs. We use these hybrid donors to introduce fluorescent protein tags into multiple loci, achieving editing efficiencies that approach those previously obtained only with much shorter ssODN donors. The principals and strategies described here are likely to translate to other systems, and should allow researchers to reproducibly and efficiently obtain both long and short precision genome edits.

## Abstract (precis)

A robust genome editing pipeline is critical to the vitality of a modern genetic laboratory. Previous studies have shown that Cas9 ribonucleoprotein (RNP)-based editing can be highly effective in *Caenorhabditis elegans*, particularly...

IN theory, CRISPR/Cas9-based genome editing enables researchers to rapidly generate designer alleles of any locus for genetic, cytological, or biochemical analyses. In practice, however, we have found that the technology remains far from routine for many users, especially in applications where long templated insertions are desired. Here, we explore the basic principles behind a robust editing pipeline. We demonstrate pronounced toxicity of ribonucleoprotein (RNP) complexes at high concentrations, and provide a strategy for optimizing RNP levels using a coinjected, easily scored reporter. Finally, we show that generating hybrid, partially single-stranded long DNA donor molecules dramatically promotes templated repair for the insertion of longer edits such as green fluorescent protein (GFP). Although, we have only tested these strategies in *Caenorhabditis elegans*, it seems likely that the principles revealed here will be relevant in other systems. The key features include:

Utilization of a DNA expression vector as a coinjection marker that controls for injection quality, permits optimization of Cas9 RNP concentration, and monitors toxicity among a cohort of progeny inheriting long DNA required for templated repair.
Employment of hybrid PCR-based donors with single-stranded homology arms for consistent, high-efficiency insertion of large constructs.

## Materials and Methods

### Strains and genetics

All the *C. elegans* strains were derived from Bristol N2 background and cultured on normal growth media(NGM) plates seeded with OP50 bacteria (Brenner 1974). Strains used in this study are listed in Supplemental Material, Table S1. Sequences of all the oligos and crRNAs are provided in File S1 and the detailed editing protocol is provided in File S2. All the reagents are available upon reasonable request.

### Data availability

The authors state that all data necessary for confirming the conclusions presented in the manuscript are represented fully within the manuscript. Supplemental material available at Figshare: https://doi.org/10.25386/genetics.7007981.

## Results

### Cas9 RNP mixtures can be toxic at high concentrations

In the course of adopting Cas9 RNP editing methodologies (Paix *et al.* 2015), we decided to monitor injection quality by adding the pRF4::*rol-6(su1006)* plasmid to the injection cocktail (Mello *et al.* 1991). We were very surprised to find that, despite giving high numbers of edited progeny, the numbers of transgenic Roller (*rol-6*) animals were greatly reduced. For example, in the course of two independent attempts to target the *vet-2* locus (a nonessential gene) we recovered a total of only 32 Rollers from 51 injected P0 worms, an average of <1 roller per injected P0. Moreover, we noted that the few surviving Roller animals obtained were often sick and sterile (data not shown), suggesting that toxicity, or off-target genome editing might cause the lack of Roller transgenics.

**Figure 1:**
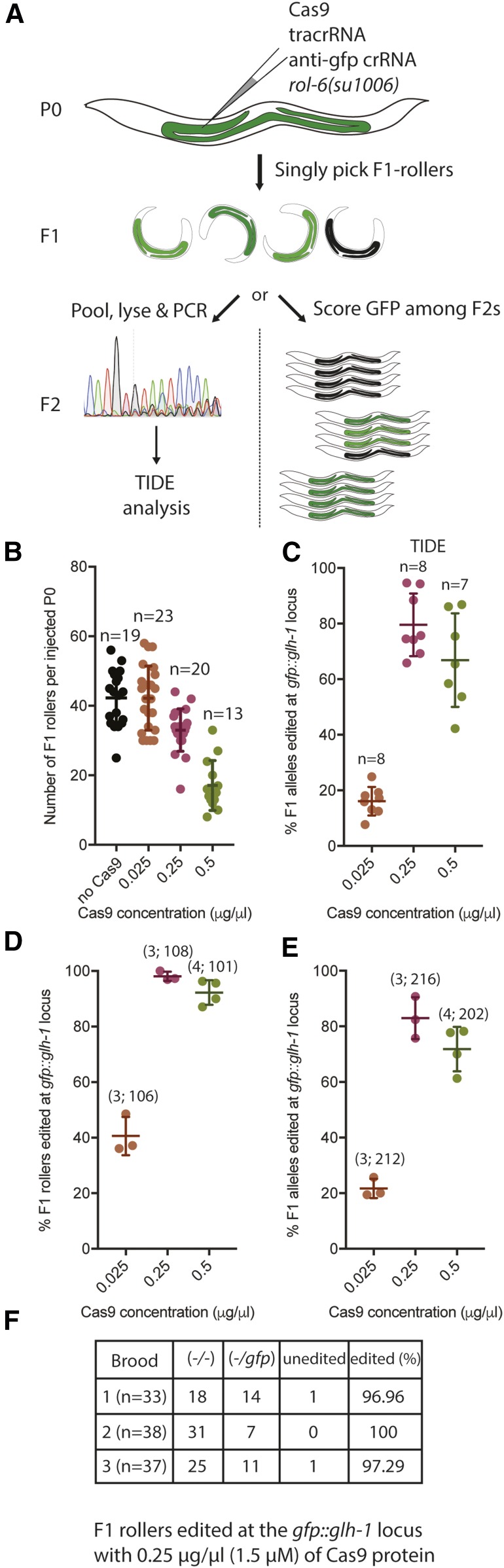
Determining optimal Cas9 RNP concentrations. (A) Schematic representation of the optimization workflow. Cas9 protein loaded with anti-GFP guide is coinjected at several concentrations with 40 ng/µl of pRF4::*rol-6(su1006)* plasmid into *gfp*::*glh-1* animals. Number of F1 Rollers segregated by each injected P0 is scored. F1 Rollers are then subjected to genotyping as a pool by TIDE analysis (left), or their F2 progeny are scored by microscopy (right). (B) Number of F1 Rollers recovered from a P0 animal injected with *rol-6(su1006)* plasmid alone, or with *rol-6(su1006)* and Cas9 RNP, at three different concentrations. Each dot represents an individual animal and (*n*) refers to the number of broods scored for each condition. Only broods containing at least one Roller were scored. (C) Percent of alleles carrying an in-del at the *gfp*::*glh-1* locus at three different Cas9 concentrations as determined by TIDE analysis. Each dot represents a pool of ≥10 F1 Rollers from one injected P0 and (*n*) refers to the number of broods scored in each condition. (D) Percentage of F1 Rollers segregating GFP-F2 negative progeny plotted *vs.* the concentration of Cas9 protein used in the injection mixture. Numbers in parentheses indicate: (number of injected P0s; number of F1 Rollers). (E) Percentage of edited *gfp*::*glh-1* alleles calculated based on numbers of homozygous and heterozygous F1 Rollers (in Figure 1D) plotted *vs.* concentration of Cas9 protein used in the injection mixture (cf. TIDE data in Figure 1C). Numbers in parentheses indicate: (number of injected P0s; number of F1 alleles). (F) A detailed breakdown of the F1 Rollers among the three broods from the 0.25 µg/µl Cas9 injection. (*n*) refers to total number of F1 Rollers. All error bars represent SD from the mean.

To address these possibilities, we performed a titration of RNP concentrations while holding the Roller DNA concentration constant. We then examined both the genome editing efficiency and the frequency of Roller transgenics among F1 progeny of the injected animals. Worms expressing the bright fluorescence marker GFP::GLH-1 (Ghanta *et al*. 2018) were coinjected with 40 ng/µl pRF4::*rol-6(su1006)* plasmid and dilutions of Cas9 RNPs loaded with an anti-gfp guide (Figure 1A). In our pilot studies we recovered very few Rollers at 2.5 µg/µl of Cas9 used in initial *C. elegans* Cas9 RNP protocols (Cho *et al.* 2013; Paix *et al.* 2015), we therefore decided to begin with a fivefold dilution, 0.5 µg/µl as a starting RNP concentration. This concentration was recently proposed by Prior *et al.* (2017). Injections using 0.5 µg/µl of Cas9 resulted in an average of 17 F1 roller progeny per injected P0 animal. Reducing the concentration by twofold, down to 0.25 µg/µl doubled the frequency of F1 Rollers to 33, while a 10-fold dilution to 0.025 µg/µl resulted in 43 F1 Roller progeny per P0 (Figure 1B). These latter two F1 roller frequencies are comparable to the rate of 42 F1 Rollers per P0 obtained when pRF4::*rol-6(su1006)* is injected alone (Mello *et al.* (1991) and Figure 1B). Taken together, these findings suggest that RNP concentrations below 0.25 µg/µl do not interfere with expression of the coinjected Roller marker gene.

We next asked how Cas9 RNP concentrations affected the indel frequency at the *gfp*::*glh-1* locus. To measure indel rates in a high throughput fashion we used the TIDE analysis pipeline, which estimates the indel rates in a mixture of PCR products (Figure 1A, left) (Brinkman *et al.* 2014). To do this we PCR amplified the *gfp*::*glh-1* locus from pools of ≥10 F1 Rollers segregated by an injected P0 worm, and subjected the mixture to Sanger sequencing and TIDE analysis. Using this approach, we found that, at 0.025 µg/µl, ∼16% of alleles carried an indel. The number of edited alleles increased to ∼80% at 0.25 µg/µl (Figure 1C), but did not increase further when the Cas9 concentration was doubled to 0.5 µg/µl, and, in fact, appeared to decline slightly to ∼67% (Figure 1C). Because GFP::GLH-1 is easily detectable in adult animals under the fluorescence-dissecting microscope, we were able to validate the TIDE results directly using microscopy (Figure 1A, right). For example, we determined that, at 0.25 µg/µl of Cas9, ∼98% of all F1 Rollers segregated GFP-negative (successfully edited) progeny (Figure 1D). Furthermore, ∼68% were homozygous, producing only GFP-negative progeny, while another ∼30% were heterozygous. Based on these numbers, we can calculate that 83% of all *gfp*::*glh-1* alleles were successfully edited at 0.25 µg/µl of Cas9 (Figure 1, E and F). These numbers correlate well with TIDE data (Figure 1C), and thus lend confidence to the calculations of the percentage of *gfp*::*glh-1* alleles cleaved at each Cas9 concentration (Figure 1, C and E). Finally, to determine the reproducibility of these findings, we repeated the injections with a previously characterized moderately efficient guide targeting the *unc-22* locus (Kim *et al.* 2014) and observed similar results (data not shown).

#### Efficient editing with synthetic single-stranded oligodeoxynucleotide donors using a Roller plasmid coinjection marker:

**Figure 2:**
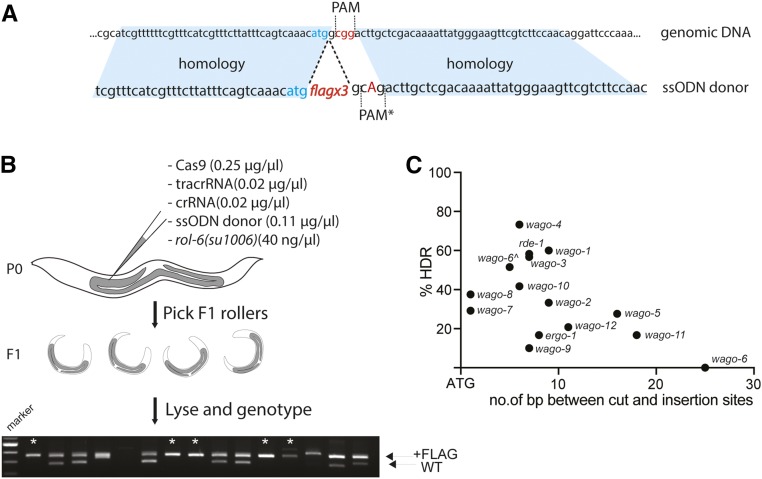
Efficient integration of 3×FLAG ssODN donor at 14 of the *C. elegans* Argonaute genes using pRF4::*rol-6(su1006)* coinjection marker. (A) Schematic of donor design for 3×FLAG insertion directly downstream of the ATG (based on Paix *et al.* 2015). Blue shading highlights homology arms, red letters indicate the PAM site, blue letters represent the START codon, capital A is the mutation introduced to disrupt the PAM site in the donor. (B) Schematic of the CRISPR protocol. Simplified injection mixture contains just the RNP components, the ssODN donor, and *rol-6* plasmid. Approximately 24 F1 Rollers from two best injection plates were cloned and genotyped. Lower band is the wild-type PCR product; upper band is upshifted due to 3×FLAG insertion. * marks putative homozygotes. (C) Efficiencies of 3×FLAG insertion plotted *vs.* distance of the guide cut site from the START codon. Detailed underlying data supplied in Table 1. Each dot represents targeting of one gene. ^ indicates the repeated attempt at targeting *sago-2* using the donor described in Figure S1.

The above findings demonstrate that Roller plasmid coinjection identifies animals that are highly likely to undergo CRISPR-induced DNA double-strand breaks. We next wished to test this methodology for achieving homology-directed repair (HDR). To do this, we decided to introduce a (3×)FLAG-affinity tag into each of the 12 worm-specific Argonautes (WAGOs) as well as two additional Argonautes: ERGO-1 and RDE-1. For each gene, we designed guides targeting the protospacer adjacent motif (PAM) site closest to the ATG start codon (without any further optimization or guide testing) [Figure 2A and File S1 for guide and synthetic single-stranded oligodeoxynucleotide (ssODN) donor sequences] (Paix *et al.* 2015). (*wago-1* and *wago-2* are highly similar near the ATG, and no specific guide could be designed; thus, one guide targeting both loci was used). Each mixture was then injected into adult N2 worms using standard worm gonadal injection methodology (Figure 2B) (Mello and Fire 1995). For each experiment, we injected ∼10 P0 animals and singled ∼24 F1 Rollers from plates segregating the most Rollers (indicative of the best injections). After producing broods, Rollers were genotyped for 3×FLAG insertions (Figure 2B, See File S2 for detailed protocol).

We were able to recover 13 out of 14 tagged strains among the first 24 F1 progeny screened by PCR from each set of injections, and, in every case, we recovered multiple independent alleles (Table 1). Although genotyping suggested that we recovered a significant number of putative F1 homozygotes (Figure 2B; asterisks), these animals were not used to establish lines. These F1 homozygotes are expected to carry two different alleles of the modified gene. For example, without additional analysis homozygous F1s could not be conclusively distinguished from *trans*-heterozygotes carrying a correct edit, over a partial or imprecise edit, or an edit that deleted one of the genotyping primer binding sites. Thus, for simplicity of the genetic analysis, independent lines were established by selecting homozygous F2s segregated by three different heterozygous F1 animals (Table S1). Accuracy of each insertion was validated by sequencing. The average success rate for precise insertion of the 3×FLAG tag ranged from 10 to 73%, and averaged ∼34% (Figure 2C and Table 1).

**Table 1:** 3×FLAG tag insertions in N-termini of 14 Argonaute genes using ssODN donors and *rol-6* coinjection marker

Locus	# F1 Rollers positive for insertion by PCR	Total# of F1 Rollers genotyped
*wago-1*	18	30
*wago-2*	10	30
*ppw-2 (wago-3)*	17	30
*wago-4*	22	30
*wago-5*	8	29
*sago-2 (wago-6)*	0	30
*sago-2 (wago-6)**^a^*	33	64
*ppw-1 (wago-7)*	7	24
*sago-1(wago-8)*	9	24
*hrde-1 (wago-9)*	3	30
*wago-10*	10	24
*wago-11*	8	48
*nrde-3 (wago-12)*	5	24
*ergo-1*	4	24
*rde-1*	14	24

Breakdown of numbers used to derive the %HDR efficiencies plotted in Figure 2C.

a Indicates the repeated attempt at targeting *sago-2* using the donor described in Figure S1.

Plotting the insertion efficiency *vs.* the distance between the Cas9-induced cut and the desired insertion site (directly after the ATG start codon; Figure 2A) we found no strong correlation up to 20 bp away (Figure 2C). The *wago-6* (*sago-2*) locus was the only outlier, likely because the nearest available cut site suitable for use with the original donor design was 27 bases away from the site of insertion. Although a number of insertions were recovered at this locus they were either out of frame or contained random DNA sequences (data not shown). The *wago-6* gene contains a second PAM site located right at the ATG start codon. This site was not used originally because the 3×FLAG donor sequence (which starts with a “G”) would not disrupt the PAM. Moreover, the alternative approach to prevent recutting of the repaired locus, mutating the guide binding site, would require introducing potentially undesirable mutations into the 5′ UTR. To solve this problem, we added an extra CCC, proline codon, to the 3×FLAG donor sequence, immediately downstream of ATG (Figure S2). Using this donor and guide we recovered *flag*::*wago-6* alleles in 52% of the F1 Roller animals analyzed.

In all of the edited strains the Roller phenotype was expressed only transiently during the F1, indicating extrachromosomal expression (Mello *et al.* 1991). These findings demonstrate the general utility of the Roller marker for identifying edited animals without introducing additional edits or undesired phenotypes into the resulting strains. In addition, these findings indicate that, as long as the desired insertion site resides within 20 bp of the cut site, ssODN donors provide for highly efficient editing.

#### Hybrid dsDNA donors promote the integration of large constructs:

High rates of HDR have been reported using PCR-generated double-stranded DNA (dsDNA) ∼1 kb-sized donors with ∼35 bp homology arms (Paix *et al.* 2015). However, we have struggled to reproduce these successes using the original or optimized protocols (data not shown). Extending the homology arms from 35 to 120 bp resulted in low (∼2%), but reproducible, levels of GFP or mCherry integration at six different loci (Table 2 and Table S2). Thus, in our hands, there was a large gap between the efficiency of templated repair using ssODNs *vs.* longer dsDNA donors.

**Table 2:** HDR efficiencies of GFP insertion with blunt-ended PCRs as donors

Locus	no. of F1 Rollers *(rol-6)*	
	Screened	GFP+
*wago-4*	54	1 (1.85%)
*sago-2 (wago-6)*	119	1 (0.84%)
*ppw-1 (wago-7)*	76	1 (1.32%)

All the donors consist of 120 bp long homology arms on both ends.

**Figure 3:**
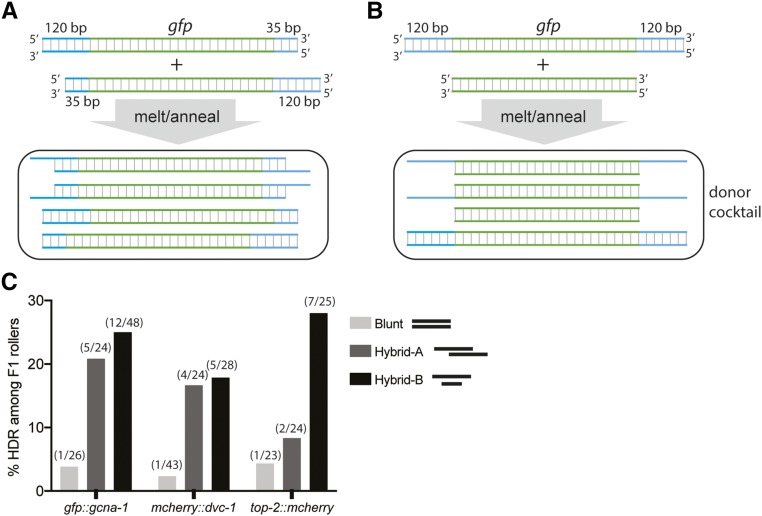
Efficient editing with long, partially single-stranded dsDNA donors. (A and B) Schematics of the strategy for generating hybrid dsDNA donor cocktail featuring molecules with ssDNA overhangs. (C) Integration efficiencies of GFP or mCherry fluorescent tags using blunt donors or hybrid dsDNA donor cocktail at diverse loci, plotted as a fraction of F1 Rollers positive for appropriate insert as detected by PCR. Numbers above each bar indicate number of insert-positive Rollers over total number of Rollers.

A recent study proposed that ssODN donors are integrated by a highly efficient single-stranded template repair pathway, while dsDNA donors rely on a less efficient HDR pathways (Richardson *et al.* 2018). We therefore wondered whether we could achieve the improved efficiency of ssDNA by employing large PCR-based donors with single-stranded overhangs. To test this idea, we generated two PCR donors to target the same locus: one with a 120-bp left homology arm and a 35-bp right homology arm, and the other with 35-bp on the left and 120-bp on the right. By mixing these donors at equimolar quantities, then melting and reannealing the mixture, we should get a mixture of four different molecules (Figure 3A), two of which have either 3′ or 5′ single-stranded overhangs. Alternatively, hybrid asymmetric PCR donors were prepared by annealing molecules with 120-bp homology arms to a PCR product containing just the insert, with no homology arms (Figure 3B). We used 200 ng/μl of blunt donor or hybrid cocktail in the optimized editing protocol (Figure 2B), and integration was scored by PCR and multiple positives were validated with sequencing across the junction as well as by microscopy. Strikingly, both types of hybrid dsDNA donor cocktails consistently yielded higher rates of accurate integration at three different loci, compared to melted and reannealed traditional blunt donors (Figure 3C). We were successful at generating N- and C-terminal fusions with GFP and mCherry tags at rates comparable to those of ssODNs, ∼20% of F1 Rollers. Hybrids between the PCR product with 120-bp homology arms and a PCR product containing just the insert, lacking arms, (Figure 3B) yielded the best precise editing rates, indicating that homology arms on the shorter product are not required to stimulate recombination. Hybrids with shorter overhangs (60 bp of homology) provided some precise insertion, but were not as effective as 120 bp arms (Table S2).

## Discussion

CRISPR/Cas9 genome editing is highly efficient in *C. elegans*, and should be accessible to investigators of all levels of experience. The protocols described here establish clear benchmarks for implementation and troubleshooting. We demonstrate efficient editing at diverse genomic loci provided that editing targets are reasonably proximal (<20 bp) to a PAM site. For short inserts (<140 bp), we find the best efficiency with ssODN donors, as was previously reported (Paix *et al.* 2015; Prior *et al.* 2017). For longer inserts, we recommend using donors that are hybrids of two asymmetric PCR products, or a hybrid of a traditional symmetric donor and the insert (Figure 3B). The detailed version of our protocol is included in File S2.

An important feature in any microinjection protocol is the inclusion of metrics that enable troubleshooting. For example, during development of basic DNA transformation protocols for *C. elegans*, it was found that poorly purified DNA, too much DNA, or even specific DNA sequences, can be toxic. Thus, the inclusion of a DNA marker such as the pRF4::*rol-6(su1006)* plasmid, that reports on the viability of progeny inheriting DNA, enables quick identification of toxic injection mixtures (Mello *et al.* 1991). The current findings suggest that, like DNA preparations, RNP mixtures can interfere with inheritance of coinjected DNA. Importantly, RNPs distribute so widely, to even hundreds of progeny, and induce oligo-mediated templated repair so efficiently (Paix *et al.* 2015), that the absence of the 20 or 30 progeny that typically inherit large coinjected DNA molecules could easily escape detection. Thus, we propose that using only indel frequency or the oligo-driven repair efficiency to monitor the activity of the editing mixture, is not sufficient. Instead, we recommend the inclusion of a plasmid-DNA-driven visible marker such as *rol-6*. Expression of this marker reports on a segment of the brood that inherits long dsDNA, and thus identifies animals that likely also inherit long dsDNA donor templates and may thus incorporate longer edits such as GFP insertions. We are not arguing that this procedure yields higher rates of editing than other (properly optimized) protocols, but rather that the current methods provide important metrics for troubleshooting, particularly when longer DNA insertions are desired.

Early *C. elegans* genome editing protocols employed DNA vectors to express all the editing components. To troubleshoot these protocols, we and others advocated using known and validated guide vectors such as *unc-22* or *dpy-10* as “co-CRISPR” markers (Arribere *et al.* 2014; Kim *et al.* 2014). Cutting at the previously validated target locus reported on DNA-driven Cas9 activity, and, because coinjected DNA vectors are generally inherited together, Mello *et al.* (1991) also reported on the viability among animals inheriting coinjected DNA repair templates (Kim *et al.* 2014). In the current protocol, we utilize RNP-driven Cas9 activity, and show that RNP activity distributes much more broadly and does not report on viability of animals receiving large DNA templates required for longer edits. Moreover, whereas vector-driven guides were often nonfunctional, with synthetic guide RNA preparations we have yet to encounter guides that do not cut the target locus. Thus, in practice, it has not been necessary to monitor the activity of each new synthetic RNA guide. Instead, we recommend a sequential test, to first monitor Cas9 RNP activity and toxicity by using a known and well-validated guide RNA (such as the GFP guide described here) and pRF4::*rol-6(su1006)* plasmid DNA. Every new batch of editing enzyme should first be tested to ensure both that editing occurs and that viable Rollers are obtained at reasonable levels (typically a few from each injected animal). These same validated conditions are then used with new guides, until or unless a problem occurs. For example, in the event that the desired edits are not obtained among the Rollers, or if Rollers are absent, then further tests will be needed to ensure that the new guide preparation is functional and not toxic. If Rollers are absent, the particular guide RNP might be toxic, perhaps cleaving an essential locus at very high efficiency. Further dilution of the guide/RNP mix until Rollers are once again observed would likely solve this problem. If editing is still not observed, one might also wish, at that point to perform a co-CRISPR test with two RNPs mixed together to monitor guide RNA toxicity. In practice, we just have not, as yet, needed to undertake these additional troubleshooting steps when using the very robust RNP methodology.

There are several additional advantages to using the pRF4 Roller expression marker for RNP-based editing. The Roller phenotype is dominant and easily scored under the light dissecting microscope. Plasmid DNA preparation is inexpensive, and injection of plasmid DNA at these concentrations results, primarily, in F1 transient expression without further inheritance in subsequent generations. Indeed, a recent study employed an *mCherry*::*myo-2* plasmid to identify ssODN-templated editing events (Prior *et al.* 2017), demonstrating the feasibility of using other plasmid-based coinjection markers for genome editing. However, we find Roller more convenient, as a fluorescence dissecting scope is not needed for scoring.

Our findings suggest that Cas9 RNP mixtures can be toxic and can eliminate F1 progeny that receive the largest amounts of coinjected long dsDNA. In this study, we tested only one source of commercially available Cas9 protein. Since RNP activity and toxicity will likely vary depending on the specific target or guide sequence, or due to variations in protein preparation or impurities, we recommend that Cas9 RNP preparations be tested routinely for optimal concentration using the simple and inexpensive *rol-6*/TIDE approach (Figure 1A).

We do not yet know how hybrid dsDNA PCR donors stimulate HDR, and it will be important to fully test the limits of this approach in terms of maximal donor length and minimal single-stranded overhangs and optimal donor concentrations. It seems likely that other modifications, such as chemical modifications to the ends of the donor molecule, may drive even greater efficiencies. The procedure for generating hybrid donors is extremely easy to implement, and we anticipate that these types of donors will also stimulate precise editing in other systems. In summary, it is now as easy to precisely edit the worm genome as it is to generate the iconic Roller transgenics first described by Mello *et al.* (1991). We strongly encourage even the total novice worm breeder to begin editing the genome of this fascinating “yeast” of metazoa.
